# Side-loading prevalence and intoxication in the night-time economy

**DOI:** 10.1016/j.abrep.2021.100403

**Published:** 2021-12-31

**Authors:** Michael P. Cameron, Peter G. Miller, Matthew Roskruge

**Affiliations:** a School of Accounting, Finance and Economics, University of Waikato, Private Bag 3105, Hamilton 3240, New Zealand; b Te Ngira - Institute for Population Research, University of Waikato, Private Bag 3105, Hamilton 3240, New Zealand; c School of Psychology, Faculty of Health, Deakin University, Australia; d School of Economics and Finance, Massey University, Private Bag 102904, Auckland 0745, New Zealand

**Keywords:** Side-loading, Pre-drinking, Intoxication, Night-time economy, New Zealand

## Abstract

Although pre-drinking has attracted considerable research interest, side-loading (any drinking occurring outside of licensed premises during a night out, and excluding drinking at home) is comparatively under-studied. In this paper, we investigate the prevalence of side-loading behaviour and intoxication in the night-time economy of Hamilton, New Zealand’s fourth-largest city. Using a street-intercept survey conducted over six nights (*n* = 469) in March and April 2019, we found that 17.5% of research participants (82/469), and 19.9% of drinkers (82/413), had engaged in side-loading. Of those engaging in side-loading, the majority did so in a car (61.0%), with smaller proportions engaging in side-loading in the street (17.1%), a carpark (12.2%), or somewhere else (13.4%). Men were significantly more likely than women to engage in side-loading behaviour (*p* = 0.001). In linear models controlling for time of the night, day of the week, and demographic variables, side-loading was not statistically significantly associated with breath alcohol content. This contrasts with pre-drinking, which was associated with statistically significantly higher breath alcohol content. Our results suggest that side-loading might not be used as a method for drinkers to enhance intoxication, but instead as a means of sustaining a target level of intoxication during an evening.

## Highlights

• A substantial minority of the ambient population in the night-time economy had engaged in side-loading.
• Unlike pre-drinking, side-loading was not statistically significantly associated with greater levels of intoxication (as measured by breath alcohol content).
• Side-loading might not be used as a method for drinkers to enhance intoxication, but instead as a means of sustaining a target level of intoxication.

## 1 Introduction

The night-time economy is an important component of contemporary urban economies, and includes leisure and hospitality activities occurring outside usual business hours. However, it is also the scene of a substantial amount of alcohol-related harm (Chikritzhs and Stockwell, 2002, Ireland and Thommeny, 1993, Miller et al., 2019), and has attracted significant research attention in recent years (e.g. Miller et al., 2019, Miller et al., 2016, Miller et al., 2013). In terms of drinking behaviour and the night-time economy, extant research has mostly focused on alcohol consumption within licensed premises (Miller et al., 2016, Miller et al., 2012) and pre-drinking (also referred to as pre-loading, pre-partying, or pre-gaming) (Hughes et al., 2011, Labhart et al., 2013, Labhart et al., 2014). The latter is defined as the consumption of alcohol before a night out or event (Borsari et al., 2007, Pedersen and LaBrie, 2007).

However, this focus on drinking before a night out, and within licensed premises during a night out, omits a potentially important alternative source of drinking during the night out. The phenomenon of ‘side-loading’ was first identified in the research literature by Forsyth (2010), who defined it as “smuggling alcohol purchased elsewhere for consumption within on-trade premises” (Forsyth, 2010, p. 32). It has since been identified using observational methods, such as in Australia (Miller et al., 2012b), but rarely directly studied. O’Rourke et al. (2016, p.147) broadened Forsyth’s definition of side-loading to “the consumption of alcohol when smuggled into licensed premises, such as pubs, bars and clubs…, or while traveling to and between licensed premises”. We note that even this definition potentially misses some important drinking behaviour, where drinkers exit a licensed premises to consume alcohol purchased elsewhere, before *returning to the same premises*. We therefore adopt an alternative definition of side-loading, being the consumption of alcohol during a night out or event, occurring at a location other than a licensed venue or a private home. This extends the definition of O’Rourke et al. (2016) to consider cases where drinkers hide alcohol outside a licensed premises, for drinking later (e.g. see Wright et al., 2014), and excludes drinking of alcohol smuggled into a licensed premises.

Despite the attention devoted to the issue of pre-drinking, side-loading has been the subject of only limited systematic study. Specifically, few studies have attempted to estimate the prevalence of side-loading or its contribution to intoxication in the night-time economy and to alcohol-related harm. In his study of 32 nightclub patrons in Glasgow, Scotland, Forsyth (2010) noted that no instances of side-loading were recorded in the weekly diaries of his research participants. He noted that opportunities for side-loading (as per his narrow definition) were limited by systematic door searches by security staff. O’Rourke (2016), based on an online survey of 2008 adults (mean age 49.7 years) in Victoria, Australia, found that 23 percent of respondents had engaged in side-loading at least once in the previous twelve months. They found that side-loading was more common among younger people, but unlike pre-drinking and back-loading (drinking after a night out, e.g. ‘after-partying’), side-loading was not statistically significantly associated with risky drinking. However, their study controlled only for demographic characteristics (age and gender), and tells us little about event-specific associations between side-loading behaviour and intoxication or alcohol-related harm. Side-loading was also noted as a considerable issue by observational staff in a comparative study of Geelong and Newcastle in Australia (Miller et al., 2012b). Key informants, observers and interviewers all noted that side-loading (which, similar to Forsyth (2010), was defined as use of hip flasks containing spirits which can be drunk straight or added to mixers) was prevalent, but there were no measures available in the study to specifically estimate harms associated with side-loading. However, venue operators clearly raised concerns about the effect of side-loading on their ability to manage patron intoxication, as well as negative impacts on their profit margins.

As noted above, we define side-loading behaviour as the consumption of alcohol during a night out or event, occurring at a location other than a licensed venue or a private home. We believe that this definition captures a range of drinking activities occurring during a night out but not controlled by bar or security staff. These activities are important to understand in terms of their potential contribution to intoxication and alcohol-related harm. Our definition omits side-loading behaviour occurring *within* licensed premises, which was the original conception of Forsyth (2010) and implied by the definition used in Miller et al. (2012b), and was included within the definition of O’Rourke (2016). As Forsyth found, drinking smuggled alcohol within licensed premises is relatively rare due to the vigilance of security staff. Moreover, our definition limits the activities considered to those that may share a common policy response, as the activities occur outside of licensed premises. Surreptitious drinking activities occurring within licensed premises require a different policy response, and are therefore best considered separately.

In this paper, we investigate the prevalence of side-loading behaviour and intoxication in the night-time economy of Hamilton, New Zealand’s fourth-largest city (population approximately 170,000). The Hamilton Central Business District (CBD) is the central entertainment, shopping, and transportation hub, and covers an area of approximately 50 city blocks. Similar to other mid-sized cities, the CBD is surrounded by mixed-use commercial and residential areas, and the resident population of the CBD itself is relatively small, with most people travelling into the CBD for entertainment, leisure activities, or shopping. In total, Hamilton had 292 on-licence alcohol outlets (including bars, night clubs, restaurants, and function venues) at the time of our study, or approximately 17.2 outlets per 10,000 population, which is slightly lower than the national average of approximately 26.5 per 10,000 population in 2014 (Cameron et al., 2016). However, the majority of these on-licence outlets are concentrated within the CBD, with relatively few suburban outlets. In 2018, there were 337 violent crimes (assault, sexual assault, abduction, or robbery) reported in the Hamilton Central area, approximately 24 percent of all of the violent crimes reported in the wider city in that year. The co-location of a substantial number of on-licence alcohol outlets and high violent crime further motivates this study and the focus on Hamilton CBD. The objectives of this paper are to explore the phenomenon of side-loading behaviour within the Hamilton CBD, and investigate the association between side-loading and intoxication.

## 2 Material and methods

We conducted a street-intercept survey in the Hamilton CBD between 9p.m. and 2:30 a.m. across three consecutive nights (Thursday, Friday, and Saturday) for one week in March 2019, and one week in April 2019. Hamilton CBD was chosen for convenience (proximity to the researcher’s institution), previous researcher experience with the location, and comparability with past research in the Hamilton night-time economy. Following an established research protocol for street-intercept surveys (Cameron et al., 2018a, Cameron et al., 2018b) and a systematic random sampling technique, every seventh pedestrian passing through a fixed location was invited to participate in the survey. The fixed location for the research was between the main taxi stand and the most popular bars in the CBD, in a well-lit and safe site. Importantly, our street-intercept method provides us with a sample that is as representative as possible of the ambient population present in the night-time economy. The systematic random sampling approach has the advantage of being simple to relay to research assistants, although sometimes the ideal of approaching every seventh person can be difficult when large groups pass and refuse participation. One disadvantage is that two members of a couple are not ever likely to be interviewed on the same night, which might exclude certain dynamics, although we don’t have any research questions that pertain to couples per se.

Data collection was undertaken by two teams of two survey assistants, overseen by a pair of senior researchers. The survey assistants (two male; two female) were thoroughly trained by the senior researchers prior to the commencement of the fieldwork. To be eligible for inclusion in the study, participants needed to be pedestrians passing through the selected intersection and aged 18 years or over. Potential participants who were judged to be too intoxicated to provide informed consent would have been excluded, although in practice this did not occur in any case. Survey assistants administered the short (approximately five-minute) survey questionnaire for each consenting eligible research participant, recording responses on paper survey forms. If further consent was given, a breathalyser test was administered at the conclusion of each interview using a recently calibrated Andatech Precision+™ breathalyser (Andatech Pty Ltd., Melbourne, Australia; accuracy to ± 0.005).

The survey collected data on demographics, pre-drinking and side-loading behaviour, and other event-specific data about the current night out (whether this is a ‘typical’ night out, intentions for the rest of the night, number of hours expected in the night-time economy. Side-loading was measured from responses to the question, “Have you had a drink at any other place (e.g. in a public place, car, carpark, or park) since you came out tonight?”. Pre-drinking was measured from responses to the question, “Did you consume any alcohol before going out tonight (e.g., in a private home or other private setting)?”. The research received ethics approval from the Waikato Management School Human Research Ethics Committee, and support from the local New Zealand Police alcohol harm reduction officer.

Of 1133 people who were approached to participate in the research, 477 interviews were undertaken – a response rate of 42.1%. After removing incomplete records, the final sample size was 469 responses, of which we have a valid breathalyser reading (including zero readings) for 451 (96.2%). Sample characteristics are summarised in the supplementary materials online, Table S1.

Data on side-loading behaviour were summarised using simple tabulations and cross-tabulations. Differences were also evaluated between students and non-students, between genders, and between pre-drinkers and non-pre-drinkers. The relationship between side-loading behaviour and intoxication (as measured by breathalyser reading) was assessed using linear regression. In our primary analysis, we include all observations, including those who were not drinking, in order to assess the relationship between side-loading and intoxication within the ambient population, rather than within the population of drinkers. We also report the results for the sub-population of drinkers within the ambient population. In both analyses, we present results with, and without, controlling for pre-drinking status. All analyses were conducted in Stata, v16.

Before presenting our results, we note a few limitations of our research. First, data collected from intoxicated research participants may be subject to greater error than data collected from those who are sober. However, we collected data that we believe are likely to be least subject to recall bias or social desirability bias, and we expect that disinhibited research participants may be more likely to respond honestly to survey questions. Moreover, we make use of an objective measure of intoxication (based on a breathalyzer reading), which further reduces measurement error problems within the data. Second, the response rate for this research was much lower than in prior similar intercept survey research in Hamilton (see Cameron et al., 2018a, Cameron et al., 2018b), which may reduce somewhat the representativeness of the sample.

## 3 Results

Approximately 90 percent of the research participants had consumed any alcohol on the day they were surveyed (for simplicity, we refer to these people as ‘drinkers’). The mean breath alcohol content at the time of interview (including those who were not drinking) was 330 mcg/L (SD 248; range 0–1170), which is well above the adult drink-driving limit of 250 mcg/L. For those who had consumed alcohol, the mean breath alcohol content was 368 mcg/L (SD 234; range 0–1170). A large majority (84.4%) of the research participants had engaged in pre-drinking, meaning that 93.8 percent of those who had consumed any alcohol that day had been pre-drinking.

Compared with pre-drinking, a substantially smaller proportion of research participants had engaged in side-loading (82/469 = 17.8% of research participants, and 82/413 = 19.9% of drinkers). Of those engaging in side-loading, the majority did so in a car (61.0%), with smaller proportions engaging in side-loading in the street (17.1%), a carpark (12.2%), or somewhere else (13.4%). ‘Other’ locations for side-loading included friends’ homes, student halls of residence, and accommodation providers (hotels or backpacker hostels).

Men were significantly more likely than women to engage in side-loading behaviour (*χ*^2^(1) = 12.059; *p* = 0.001), and pre-drinkers were slightly significantly more likely to engage in side-loading than non-pre-drinkers (*χ*^2^(1) = 3.785; *p* = 0.052). Women were significantly more likely than men to engage in side-loading in a carpark (*χ*^2^(1) = 4.228; *p* = 0.040), while pre-drinkers were significantly less likely to engage in side-loading in a location other than a car, carpark, or the street (*χ*^2^(1) = 5.712; *p* = 0.017).

Linear regression results for the relationship between side-loading and intoxication (as measured by breath alcohol content) are reported in Table 1. The results for four models are reported. In Models (1) and (2), the full sample is included. Model (2) controls for pre-drinking behaviour, while Model (1) does not. Models (3) and (4) repeat Models (1) and (2) respectively, but limit the sample to the subpopulation of those who had been drinking on the night they were surveyed. All models control for gender, age (and age-squared), student status, whether the respondent was local or a visitor, day of the week, month, and time of survey (full regression results are available in the supplementary materials online, Table S2). Separate models by gender showed no significant differences from the combined models (results are available in the supplementary materials online, Tables S3 and S4).

**Table 1:** Linear regression of factors associated with breath alcohol content.

**Variables**	**Model (1)**	**Model (2)**	**Model (3)**	**Model (4)**
Side-loading status	0.065	0.007	0.004	−0.011
(1 = yes)	[41.342]	[4.140]	[2.410]	[-6.095]
	(25.297)	(23.811)	(24.560)	(23.685)
Pre-drinking status		0.445***		0.257***
(1 = yes)		[258.050]		[167.721]
		(27.397)		(35.068)
Observations	446	446	401	401
R-squared	0.125	0.279	0.099	0.151

N.B. Coefficients are reported first as standardised betas; Unstandardised coefficients are reported in square brackets; Robust standard errors are reported in parentheses; *** p < 0.01, ** p < 0.05, * p < 0.1; All models control for gender, age (and age-squared), student status, whether the respondent was local or a visitor, day of the week, month, and time of night (full regression results are available in the supplementary materials online, Table S2).

Perhaps surprisingly, engaging in side-loading behaviour is not statistically significantly associated with breath alcohol content in any of the models. The coefficient is small, and the sign is inconsistent across models. In contrast, pre-drinking is statistically significantly associated with higher breath alcohol content in both Model (2) and Model (4), and the size of the coefficient is large – pre-drinking is associated with 258 mcg/L higher breath alcohol content when considering the full sample, and 167 mcg/L higher breath alcohol content in the sample of drinkers.

## 4 Discussion and conclusions

Side-loading has the potential to contribute to increasing levels of intoxication among bar patrons and increasing alcohol-related harm in the night-time economy. Despite this, side-loading has not been subject to systematic study to the same extent as pre-drinking. We found that a substantial minority of the ambient population in the night-time economy of Hamilton had engaged in side-loading, with a higher prevalence of side-loading among men. However, we also found that side-loading was not associated with greater levels of intoxication (as measured by breath alcohol content). This lack of association held both with and without controlling for pre-drinking behaviour. In contrast, pre-drinking was associated with significantly higher levels of intoxication.

There are a number of reasons why side-loading behaviour may not be associated with intoxication to the same degree as pre-drinking. It is notable that the Hamilton CBD is a liquor ban area, where consumption of alcohol in public spaces is banned and subject to enforcement by the police. Thus, for many of the locations in which side-loading occurred, including the street and car parks, this drinking is illegal and comes with the risk of penalties and/or the confiscation of the alcohol. This situation does not lend itself to prolonged periods of side-loading, in contrast with pre-drinking, and this might limit the effect of side-loading on measured levels of intoxication. This may also explain gender differences, as men may be more willing to accept risk (Borghans et al., 2009), including the risk of penalties for drinking in the liquor ban area. On the other hand, side-loading is likely to be conducted in a more rapid fashion in order to avoid detection. Nevertheless, as the overall effect on intoxication was not significant, it is possible that side-loading is not used as a method for drinkers to enhance intoxication, but may merely be a means of sustaining a target level of intoxication (Measham, 2006). The lack of association with intoxication that we find in our research is similar to the results of O’Rourke (2016), who found no association between side-loading behaviour and risky drinking, albeit in a population-level survey. As drinkers may use side-loading as a substitute for purchasing drinks at a bar or night club, the number of drinks consumed in total (and hence intoxication) may not change, only the location of the drinking. This potential role of side-loading as a substitute may arise through the same price-disparity mechanism that contributes to pre-drinking behaviour, i.e. the large disparity in the price of alcohol between on-licence outlets (night clubs, bars, or restaurants) and off-licence outlets (bottle stores, or supermarkets) (e.g. McCreanor et al., 2015). To further understand how side-loading contributes (or not) to the level of intoxication over the course of a night, future research should seek to collect longitudinal data from individual drinkers (e.g. Kuntsche and Labhart, 2012, Labhart et al., 2017), as well as further investigating the role of and motivations for side-loading as a potential substitute for in-bar drinking.

We found that side-loading was not as prevalent as pre-drinking. This may be because, by definition, side-loading requires patrons to leave a licensed premises, removing them from ‘the action’ and access to entertainment and those with whom they may wish to socialise (unless their companions leave also). Leaving a heavily-patronised venue may also necessitate a period of waiting in line for re-entry. These factors increase the psychological costs associated with side-loading, and reduce its appeal. Lockouts and door charges may also act to reduce side-loading behaviour.

Greater understanding of the motivations for side-loading behaviour remains elusive. Unfortunately, we did not include questions on motivations for side-loading, nor directly investigate the factors that may reduce its prevalence. Future research should collect more complete data on the range of side-loading behaviours, and the motivations underlying the range of side-loading activities, including the location and how much is consumed during side-loading. It would also be useful to investigate whether side-loaders engage in one, or multiple, side-loading episodes during a night out. Moreover, similar to Cameron et al. (2020), for policy purposes it would be useful to identify when and where alcohol for side-loading is purchased, and where and how it is stored or stashed while the side-loader is in licensed premises. We note these as key opportunities and questions to be addressed in future research.

## Contributors

MC and MR acquired the funding, conducted the fieldwork and data analysis, and wrote the original draft. MC, PM, and MR all contributed to conceptualization and research design, interpretation of results, and review and editing of the final paper.

## Role of funding sources

This research was funded by Health Promotion Agency/Te Hiringa Hauora (HPA). The funder had no involvement in data collection, analysis, or interpretation of the data, or writing of the manuscript. The funder provided some initial feedback on study design, but the authors retained full control over all aspects of the study.

## Declaration of Competing Interest

The authors declare the following financial interests/personal relationships which may be considered as potential competing interests: Michael P. Cameron is a District Licensing Committee member and commissioner in New Zealand, and has acted as an unpaid expert witness for local authorities before the Alcohol Regulatory and Licensing Authority. Peter G. Miller receives funding from Australian Research Council and Australian National Health and Medical Research Council, grants from NSW Government, National Drug Law Enforcement Research Fund, Foundation for Alcohol Research and Education, Cancer Council Victoria, Central Australian Aboriginal Congress, Northern Territory government, Australian Rechabites Foundation, Northern Territory Primary Health Network, Lives Lived Well, Queensland government and Australian Drug Foundation, travel and related costs from Queensland Police Service, Northern Territory government, Queensland Office of Liquor Gaming and Racing and the Australasian Drug Strategy Conference. He has acted as a paid expert witness on behalf of a licensed venue and a security firm.
