# Supplementary material for: Side-loading prevalence and intoxication in the night-time economy
Source: Addict Behav Rep. 2021 Dec 31;15:100403. doi: 10.1016/j.abrep.2021.100403 (PMC8739460; doi:10.1016/j.abrep.2021.100403)
Supplement: Supplementary Data 1 [file mmc1.docx]

***Table S1: Summary statistics (n=469)***

| **Variable** | **Mean/Percentage** | **S.D.** | **Maximum** | **Minimum** |
| --- | --- | --- | --- | --- |
| *Dependent variables* |  |  |  |  |
| Breath alcohol content (mcg/L) | 330 | 248 | 0 | 1170 |
| Self-reported intoxication (0-10 scale) | 5.04 | 2.48 | 0 | 10 |
|  |  |  |  |  |
| *Individual-specific variables* |  |  |  |  |
| Gender (1 = male) | 66.3% |  |  |  |
| Age | 23.8 | 7.22 | 18 | 71 |
| Local (1 = yes) | 67.8% |  |  |  |
| Student (1 = yes) | 44.1% |  |  |  |
|  |  |  |  |  |
| *Evening-specific variables* |  |  |  |  |
| Week of survey – Week 1 (March) | 51.6% |  |  |  |
| Week of survey – Week 2 (April) | 48.4% |  |  |  |
| Night of survey - Thursday | 10.5% |  |  |  |
| Night of survey - Friday | 36.5% |  |  |  |
| Night of survey - Saturday | 53.1% |  |  |  |
| Time of survey | 12:27 a.m. | N/A | 9:15 p.m. | 2:59 a.m. |
|  |  |  |  |  |
| *Event-specific variables* |  |  |  |  |
| Consumed any alcohol (1 = yes) | 90.0% |  |  |  |
| Pre-drinker (1 = yes) | 84.4% |  |  |  |
| Number of hours drinking | 4.86 | 3.18 | 0.02 | 15 |
| Typical night out - Yes | 55.8% |  |  |  |
| Typical night out - No, usually  smaller | 30.7% |  |  |  |
| Typical night out - No, usually bigger | 13.5% |  |  |  |

***Table S2: Linear regression of factors associated with breath alcohol content (in full)***

| **Variables** | **Model (1)** | **Model (2)** | **Model (3)** | **Model (4)** |
| --- | --- | --- | --- | --- |
| Side-loading status | 0.065 | 0.007 | 0.004 | -0.011 |
| (1 = yes) | [41.342] | [4.140] | [2.410] | [-6.095] |
|  | (25.297) | (23.811) | (24.560) | (23.685) |
| Pre-drinking status |  | 0.445*** |  | 0.257*** |
| (1 = yes) |  | [258.050] |  | [167.721] |
|  |  | (27.397) |  | (35.068) |
| Identifies as male | -0.015 | 0.038 | 0.031 | 0.054 |
| (1 = yes) | [-7.691] | [19.960] | [14.866] | [25.956] |
|  | (23.345) | (21.447) | (23.631) | (23.258) |
| Age | 0.407* | 0.633*** | 0.595*** | 0.719*** |
|  | [14.069] | [21.842] | [20.119] | [24.299] |
|  | (7.403) | (7.228) | (7.628) | (7.620) |
| Age-squared | -0.411** | -0.577*** | -0.585*** | -0.666 |
|  | [-0.206] | [-0.289] | [-0.286] | [-0.325] |
|  | (0.100) | (0.099) | (0.100) | (0.100) |
| Is a current student | -0.030 | -0.032 | -0.027 | -0.031 |
| (1 = yes) | [-14.982] | [-15.698] | [-12.409] | [-14.178] |
|  | (23.237) | (20.762) | (23.621) | (22.812) |
| Lives locally | 0.048 | 0.085** | 0.068 | 0.092** |
| (1 = yes) | [25.275] | [45.169] | [33.624] | [45.564] |
|  | (23.670) | (21.367) | (23.575) | (23.090) |
| Friday night survey | 0.211*** | 0.101 | 0.261*** | 0.169* |
| (1 = yes) | [107.502] | [51.502] | [125.023] | [80.810] |
|  | (41.269) | (39.005) | (44.968) | (44.101) |
| Saturday night survey | 0.166** | 0.038 | 0.207** | 0.100 |
| (1 = yes) | [81.715] | [18.854] | [95.502] | [46.094] |
|  | (38.850) | (36.947) | (42.244) | (42.160) |
| Week of survey | 0.003 | -0.028 | -0.010 | -0.035 |
| (1 = 2^nd^ week surveying) | [1.352] | [-13.908] | [-4.752] | [-16.189] |
|  | (22.270) | (20.481) | (22.559) | (22.384) |
| Hour of survey | 0.307*** | 0.173*** | 0.228*** | 0.172*** |
| (24hr) | [49.856] | [28.119] | [36.987] | [27.999] |
|  | (7.584) | (7.236) | (8.387) | (8.195) |
| Constant | -1,190.431*** | -954.126*** | -963.134*** | -924.421*** |
|  | (219.797) | (203.572) | (232.050) | (226.421) |
|  |  |  |  |  |
| Observations | 446 | 446 | 401 | 401 |
| R-squared | 0.125 | 0.279 | 0.099 | 0.151 |
| F-statistic (DF) | 8.25 (10, 435)  *p* < 0.001 | 20.23 (11, 446)  *p* < 0.001 | 5.82 (10, 390)  *p* < 0.001 | 7.83 (11, 389)  *p* < 0.001 |
|  |  |  |  |  |

N.B. Coefficients are reported first as standardised betas (except the constant term); Unstandardised coefficients are reported in square brackets; Robust standard errors are reported in parentheses; *** p<0.01, ** p<0.05,
* p<0.1

***Table S3: Linear regression of factors associated with breath alcohol content for those identifying as male only***

| **Variables** | **Model (1)** | **Model (2)** | **Model (3)** | **Model (4)** |
| --- | --- | --- | --- | --- |
| Side-loading status | 0.092* | 0.029 | 0.022 | 0.009 |
| (1 = yes) | [55.825] | [17.746] | [12.135] | [5.126] |
|  | (30.461) | (28.312) | (29.290) | (28.121) |
| Pre-drinking status |  | 0.448*** |  | 0.250*** |
| (1 = yes) |  | [259.410] |  | [161.185] |
|  |  | (32.757) |  | (41.369) |
| Age | 0.535** | 0.725*** | 0.785*** | 0.880*** |
|  | [17.429] | [23.620] | [24.777] | [27.774] |
|  | (8.312) | (8.400) | (8.711) | (8.888) |
| Age-squared | -0.545** | -0.675*** | -0.783*** | -0.835*** |
|  | [-0.249] | [-0.309] | [-0.345] | [-0.367] |
|  | (0.110) | (0.113) | (0.112) | (0.115) |
| Is a current student | -0.009 | -0.001 | 0.005 | 0.006 |
| (1 = yes) | [-4.635] | [-0.359] | [2.440] | [3.037] |
|  | (28.964) | (25.541) | (29.618) | (28.420) |
| Lives locally | 0.052 | 0.093* | 0.087 | 0.109* |
| (1 = yes) | [27.857] | [49.804] | [43.381] | [54.263] |
|  | (29.711) | (26.837) | (29.906) | (29.244) |
| Friday night survey | 0.155 | 0.033 | 0.227 | 0.114 |
| (1 = yes) | [80.744] | [17.000] | [110.183] | [55.520] |
|  | (59.844) | (55.361) | (67.172) | (65.034) |
| Saturday night survey | 0.105 | -0.024 | 0.160 | 0.044 |
| (1 = yes) | [53.051] | [-12.245] | [75.732] | [20.950] |
|  | (56.469) | (52.957) | (63.345) | (62.540) |
| Week of survey | -0.007 | -0.051 | -0.061 | -0.082 |
| (1 = 2^nd^ week surveying) | [-3.581] | [-25.725] | [-28.780] | [-38.861] |
|  | (29.198) | (26.499) | (30.064) | (29.290) |
| Hour of survey | 0.297*** | 0.171*** | 0.178*** | 0.141** |
| (24hr) | [49.428] | [28.420] | [30.099] | [23.933] |
|  | (9.481) | (8.811) | (10.943) | (10.450) |
| Constant | -1,221.993*** | -948.967*** | -838.114*** | -829.329*** |
|  | (268.274) | (246.125) | (297.017) | (288.638) |
|  |  |  |  |  |
| Observations | 297 | 297 | 262 | 262 |
| R-squared | 0.133 | 0.290 | 0.099 | 0.151 |
| F-statistic (DF) | 5.51 (9, 287)  *p* < 0.001 | 15.07 (10, 286)  *p* < 0.001 | 3.36 (9, 252)  *p* < 0.001 | 4.69 (10, 251)  *p* < 0.001 |
|  |  |  |  |  |

N.B. Coefficients are reported first as standardised betas (except the constant term); Unstandardised coefficients are reported in square brackets; Robust standard errors are reported in parentheses; *** p<0.01, ** p<0.05,
* p<0.1

***Table S4: Linear regression of factors associated with breath alcohol content for those identifying as female only***

| **Variables** | **Model (1)** | **Model (2)** | **Model (3)** | **Model (4)** |
| --- | --- | --- | --- | --- |
| Side-loading status | -0.026 | -0.078* | -0.058 | -0.083* |
| (1 = yes) | [-20.163] | [-60.838] | [-42.150] | [-60.464] |
|  | (33.758) | (35.263) | (33.683) | (34.725) |
| Pre-drinking status |  | 0.447*** |  | 0.286*** |
| (1 = yes) |  | [265.297] |  | [190.525] |
|  |  | (48.376) |  | (65.723) |
| Age | -0.350 | 0.221 | -0.120 | 0.249 |
|  | [-15.029] | [9.478] | [-5.038] | [10.464] |
|  | (30.104) | (26.825) | (29.525) | (28.355) |
| Age-squared | 0.355 | -0.151 | 0.157 | -0.175 |
|  | [0.260] | [-0.111] | [0.112] | [-0.125] |
|  | (0.516) | (0.458) | (0.508) | (0.482) |
| Is a current student | -0.087 | -0.097 | -0.100 | -0.108 |
| (1 = yes) | [-40.163] | [-44.878] | [-44.567] | [-47.999] |
|  | (40.098) | (35.707) | (39.997) | (38.247) |
| Lives locally | 0.030 | 0.060 | 0.013 | 0.048 |
| (1 = yes) | [15.609] | [31.065] | [6.666] | [23.628] |
|  | (39.432) | (35.406) | (37.681) | (37.272) |
| Friday night survey | 0.337*** | 0.256** | 0.354*** | 0.298** |
| (1 = yes) | [164.204] | [124.199] | [164.263] | [138.408] |
|  | (51.230) | (51.572) | (52.403) | (54.604) |
| Saturday night survey | 0.286** | 0.162 | 0.309*** | 0.211* |
| (1 = yes) | [130.783] | [74.168] | [135.225] | [92.627] |
|  | (51.905) | (48.635) | (52.838) | (54.126) |
| Week of survey | 0.051 | 0.059 | 0.103 | 0.084 |
| (1 = 2^nd^ week surveying) | [24.053] | [27.860] | [46.276] | [37.858] |
|  | (36.683) | (32.442) | (36.040) | (35.325) |
| Hour of survey | 0.335*** | 0.175** | 0.314*** | 0.209** |
| (24hr) | [51.823] | [27.012] | [47.076] | [31.328] |
|  | (13.469) | (13.390) | (13.995) | (14.716) |
| Constant | -859.472* | -786.775* | -867.655* | -844.970* |
|  | (441.609) | (410.682) | (440.560) | (433.689) |
|  |  |  |  |  |
| Observations | 149 | 149 | 139 | 139 |
| R-squared | 0.200 | 0.344 | 0.201 | 0.265 |
| F-statistic (DF) | 5.35 (9, 139)  *p* < 0.001 | 12.36 (10, 138)  *p* < 0.001 | 5.07 (9,1293)  *p* < 0.001 | 7.91 (10, 128)  *p* < 0.001 |
|  |  |  |  |  |

N.B. Coefficients are reported first as standardised betas (except the constant term); Unstandardised coefficients are reported in square brackets; Robust standard errors are reported in parentheses; *** p<0.01, ** p<0.05,
* p<0.1
